# Horn growth appears to decline under intense trophy hunting, but biases in hunt data challenge the interpretation of the evolutionary basis of trends

**DOI:** 10.1111/eva.13207

**Published:** 2021-05-05

**Authors:** Michael B. Morrissey, Anne Hubbs, Marco Festa‐Bianchet

**Affiliations:** ^1^ School of Biology University of St Andrews St Andrews UK; ^2^ Alberta Environment and Parks Rocky Mountain House Alberta Canada; ^3^ Département de Biologie Université de Sherbrooke Sherbrooke Quebec Canada

**Keywords:** artificial selection, bighorn sheep, evolution, trophy hunting, Wildlife Management

## Abstract

A recent article in *Evolutionary Applications* by LaSharr et al. reports on trends in the size of horns of bighorn sheep (*Ovis canadensis*) throughout much of the species’ range. The article concludes that there are “... stable or increasing trends in horn growth over nearly 3 decades in the majority of hunt areas throughout the western U.S. and Canada.” However, the article equates nonsignificance of predominantly negative trends in the areas with the most selective harvest as evidence for the null hypothesis of no trends and also fails to consider well‐known and serious biases in the use of data collected in size‐regulated hunts. By applying meta‐analysis to the estimates reported by LaSharr et al., we show that there has been a pervasive overall trend of declining horn sizes in Alberta, where the combination of horn size‐based legality, combined with unrestricted hunter numbers are understood to generate the greatest selective pressures. Given the nature of the biases in the underlying data, the magnitudes of the trends resulting from our re‐analysis of LaSharr et al.'s (*Evolutionary Applications*, 2019, **12**, **1823**) trend estimates are probably underestimated.

## 1 INTRODUCTION

Human actions have the potential to generate artificial selection and evolution in wild populations (Hendry et al., 2017; Stockwell et al., 2003), and one of the most important agents of anthropogenic selection can be harvest (Fugère & Hendry, 2018). In a recent article in *Evolutionary Applications*, LaSharr et al. (2019) argue that horn size in bighorn sheep (*Ovis canadensis*) throughout their range is generally stable or increasing, largely irrespective of the degree of selectivity of trophy hunting in different regions. Harvest of bighorn sheep rams is limited throughout the species’ range by varying combinations of limited entry hunt systems and a definition of minimum horn size of harvestable (“legal”) rams.

The degree to which such regulations cause artificial selection against large horn size will be determined by both the nature of the size‐dependent harvest and by the overall harvest rate. In most US jurisdictions, harvest rates are relatively low, and licence numbers are typically controlled by limited entry hunts. As such, even though some jurisdictions impose minimum thresholds on the horn size, below which harvest is illegal, low overall harvest rates probably mean that selection in most of the US part of the range is low. In contrast, in the Canadian province of Alberta, where approximately 15% of bighorn sheep occur (Larkins 2012), selection is likely to be much stronger, and much more directly dictated by minimum size requirements. In most of Alberta, rams may not be harvested until a legislated degree of horn curl is reached, and harvest rates of legal individuals may be very high as unlimited numbers of licences are available for resident hunters in most management areas. As such, LaSharr et al. (2019) surmise that harvest pressure on “legal” rams is likely higher in Alberta than elsewhere, an interpretation with which we agree. However, despite the expectation of strong selection in Alberta, LaSharr et al. (2019) conclude that the data show “... stable or increasing trends in horn growth over nearly 3 decades in the majority of hunt areas throughout the western U.S. and Canada.”

LaSharr et al. (2019) note that in Alberta “Age‐specific horn size declined in 44% of hunt areas where harvest was regulated solely by morphological criteria”, yet conclude that “phenotypic consequences are not a foregone conclusion in the face of selective harvest; over half of the hunt areas with highly selective and intensive harvest did not exhibit age‐specific declines in horn size”. While basic theory of the evolution of quantitative traits (Walsh & Lynch, 2018) recognizes that responses to selection are not a foregone conclusion, the tempering of significant declines in 44% of areas with a notion that more than half of areas in Alberta show stable horn size is problematic. Sample size is greatly diminished in any one area, reducing statistical power. Nearly all areas have negative trend estimates.

We present alternative analyses of LaSharr et al.'s results. First, we conduct a re‐analysis of estimates of trends in horn size, using established meta‐analytic techniques, to avoid equating nonsignificance with evidence for the null hypothesis. We then discuss statistical artefacts that likely dampen trends of declining horn size in harvest data.

## 2 NONSIGNIFICANCE VS. SUPPORT FOR THE NULL HYPOTHESIS

LaSharr et al. (2019) report that, after accounting for environmental variables and demography, the regressions of trophy scores (combinations of linear measurements in which length is heavily weighted) on time were nonsignificant or significantly positive in most management areas. To conclude from a statistically nonsignificant result that there is no meaningful effect would require an analysis sufficiently powerful to exclude the possibility that a substantive effect exists.

To see how LaSharr et al.'s (2019) estimates suffer from low power, consider one of the areas with the largest sample size, Kananaskis‐North in Alberta. The data (Tables S1 and S2 of LaSharr et al., 2019) included measurements from 344 rams over 24 years. The estimated slope of the regression of horn size on year was 0.01 cm/year, and the 95% confidence interval was −0.12 to 0.14 cm/year. This nonsignificant result is associated with a range of rates of change that encompases increases or decreases of approximately one cm per decade, or more than 2 cm over the available time series. Is this range of changes small enough that the population can be characterized as stable? Particularly if many males are harvested the year they become legal, 2 cm could be the difference between legally harvestable, or not.

## 3 HIERARCHICAL MODEL‐BASED ANALYSIS OF HORN TRENDS

Statistical noise arising from small sample size will (a) make small effects likely nonsignificant, and (b) inflate variability from one estimate to the next. While estimated regressions of horn size on time in each area are highly uncertain, it is possible to estimate the average trends, and the variability of trends among areas, with a useful amount of precision.

We applied a mixed effects model to LaSharr et al.'s (2019) trend estimates as reported in their supplemental tables following

(1a) $${\hat{\beta }}_{i}=\beta_{i}+m_{i}$$

(1b) $$\beta_{i}∼N\left(\mu_{\beta },\sigma_{\beta }^{2}\right)$$

(1c) $$m_{i}∼N\left(0,{\mathrm{SE}}_{i}^{2}\right),$$

where ${\hat{\beta }}_{i}$ is the estimated slope for area i and ${\mathrm{SE}}_{i}$ its standard error. $\beta_{i}$ is the (unknown) true regression slopes, and $m_{i}$ is the estimation errors of those slopes (also unknown). The model integrates over the uncertainty in the measurement errors $m_{i}$, whose distribution is given by their corresponding standard errors (${\mathrm{SE}}_{i}$). We can estimate the average of true slopes ($\mu_{\beta }$) and their variances ($\sigma_{\beta }^{2}$). Equation (1a), (1b), (1c) is a random effect meta‐analysis (Koricheva et al., 2013).

We applied the mixed effects meta‐analytic model (Equation (1a), (1b), (1c)) to data from management areas in Alberta, implemented as a Bayesian mixed model using the R package MCMCglmm (Hadfield, 2010), applying diffuse Gaussian and inverse gamma priors (Gelman & Hill, 2007) for the model intercept (the mean slope, $\mu_{\beta }$), and the residual variance (the variance of slopes, $\sigma_{\beta }^{2}$), respectively. We estimated the mean and variance of slopes of (a) horn size, (b) horn size standardized to age seven and (c) horn size standardized to age seven accounting for environmental effects. The raw data are the trend estimates in Table S1 of LaSharr et al. (2019) for uncorrected horn size and in Table S2 for the two measures standardized to age seven. LaSharr et al.'s supplemental tables give 95% confidence intervals for all slopes; we calculated standard errors for each estimate as one quarter of the difference between the upper and lower limits of each confidence interval. In most hunt areas in Alberta, size‐based harvest regulations have been consistent over the study period. In the Westcastle‐Yarrow area, the legal harvest criterion was changed from 4/5 curl to full curl in 1996, forcing a legislated strong increase in mean horn size among harvested individuals over time. We therefore conducted all analyses with and without the Westcastle‐Yarrow area.

The meta‐analytic model estimates a decline for all three types of horn size trend, across all areas within Alberta with consistent regulations (Table 1). We focus on predicted horn sizes for age seven accounting for environmental variables, which is most relevant to the potential contribution of evolution to changing horn size. These analyses are the basis of LaSharr et al.'s key second table and third figure. The average change in predicted horn size is $\mu_{\beta }$ = −0.08 cm per year (95% CI: −0.12 to −0.05 cm/year). Slopes vary among areas with $\sigma_{\beta }^{2}$ = 0.0026 (95% CI: 2 × 10^−4^ to 0.0072). From this mean and variance of slopes, we estimate the proportion of areas in Alberta where the temporal trend in horn size is negative, as

(2) $$P\;\left[\beta <0\right]=\Phi \;\left(0,\mu_{\beta },\sigma_{\beta }^{2}\right),$$

where $\Phi \;\left(0,\mu_{\beta },\sigma_{\beta }^{2}\right)$ is a cumulative normal distribution function with mean $\mu_{\beta }$ and variance $\sigma_{\beta }^{2}$, evaluated at 0. Applying Equation (2) to the estimated mean and variance of regression slopes in Alberta, we estimate that 95% (95% CI: 79–100) of areas experience a decline.

**TABLE 1:** Estimated temporal trends in (a) horn size of harvested bighorn sheep, regardless of age, (b) horn size corrected to age seven, using estimated growth curves, and (c) age seven‐corrected size measurements, controlling for environmental variables

Management area	$\mu_{\beta }$	$\sigma_{\beta }$	P(β<0)
(a) Horn size			
Alberta (all)^a^	−0.04 (−0.12 to 0.03)	0.13 (0.07–0.20)	0.63 (0.42–0.85)
Alberta (consistent)	−0.06 (−0.11 to −0.01)	0.07 (0.03–0.12)	0.82 (0.60–1.00)
USA	−0.02 (−0.08 to 0.03)	0.18 (0.13–0.23)	0.55 (0.42–0.67)
(b) Horn size, corrected to age 7			
Alberta (all)	−0.09 (−0.13 to −0.05)	0.05 (0.02–0.09)	0.93 (0.79–1.00)
Alberta (consistent)	−0.09 (−0.13 to −0.06)	0.05 (0.02–0.08)	0.96 (0.84–1.00)
USA	−0.02 (−0.07 to 0.01)	0.11 (0.07–0.13)	0.59 (0.46–0.73)
(c) Horn size, corrected to age 7, controlling for environment			
Alberta (all)	−0.08 (−0.12 to −0.04)	0.05 (0.02–0.09)	0.93 (0.77–1.00)
Alberta (consistent)	−0.08 (−0.12 to −0.05)	0.05 (0.02–0.09)	0.95 (0.79–1.00)
USA	−0.02 (−0.07 to 0.01)	0.13 (0.10–0.16)	0.57 (0.45–0.69)

Note

Analyses are conducted for three subsets of the data: first, for all of Alberta, second, for management areas in Alberta that had consistent size‐based harvest regulations throughout the study, and third, for US jurisdictions. Hierarchical model summaries include $\overset{‐}{\beta }$, the average slope across management areas, $\sigma_{\beta }$, the standard deviation of slopes across management areas, and P(β<0), the proportion of management areas experiencing a declining trend. All estimates are posterior means with 95% credible intervals in parentheses.

^a^ One management area in this subset is expected to have a substantially positively biased trend as a result of a change to the size‐based harvest regulations during the course of the study. This bias is likely to be manifested primarily in the raw size data (part a); standardizations of size measurements to age seven should at least eliminate the bias associated with the management change for parts (b) and (c) because these analyses attempt to disambiguate effects of age and size at age.

The US jurisdictions likely experience weaker selective hunting than Alberta. We obtained much smaller estimates of overall decline, with CIs that substantially overlap zero. For age‐corrected trends accounting for environmental variables, the slope across US areas averages −0.02 cm per year (95% CI: −0.07 to 0.01). Combined with the estimate of variability (0.02; 95% CI: 0.01–0.03), our estimate of the proportion of US areas with declines in horn size is 57% (95% CI: 45–69).

**FIGURE 1:**
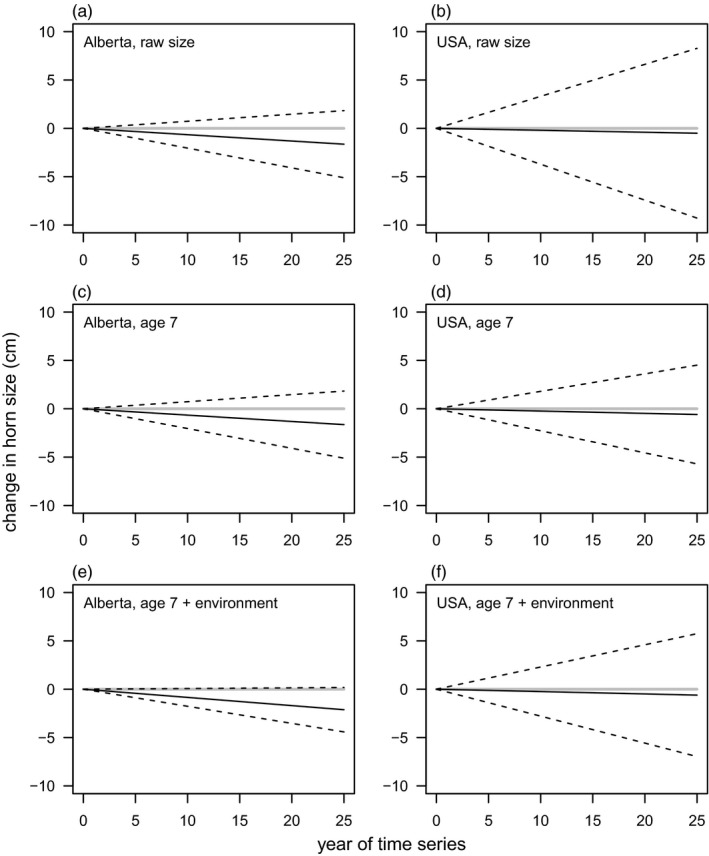
Estimated distributions of temporal trends in bighorn sheep horn size in Alberta (a,c,e) and in American jurisdictions (b,d,f). Trends are depicted as solid black lines, showing changes from an arbitrary initial value of zero. Grey lines have a slope of zero. Upper and lower boundaries (dashed) lines represent the hierarchical model's best estimate of the biological variability among management areas (within Alberta and the USA, separately) in temporal trend by depicting the limits of 95% of the distribution of among management trends. Uncertainty in the means and standard deviations of temporal trends are reported as 95% credible intervals in Table 1

The proportion of areas experiencing a decline does not measure the magnitudes of trends. The mean and among‐area variability in trends, inferred from the hierarchical model analyses, are depicted in Figure 1. While jurisdictions in the USA do not show consistent declines, variability among management areas is greater than in Alberta. When declines occur, they can be of similar magnitude to trends in Alberta (Figure 1). Within all jurisdictions that have declined, those declines are about 5% of mean horn size.

In addition the main question of whether or not there are consistent trends in horn size in Alberta and in the United States, the difference in the average slope between these two groups of areas is also of interest. We fitted a model to directly estimate this difference, as

(3a) $${\hat{\beta }}_{i}=\beta_{\mathrm{US}}+\delta_{\beta }\phi_{\mathrm{AB}}+m_{i}$$

(3b) $$\beta_{i}∼N\left(\mu_{\beta },\sigma_{\mathrm{loc}}^{2}\right)$$

(3c) $$m_{i}∼N\left(0,{\mathrm{SE}}_{i}^{2}\right).$$

Notation is as for Equation (1a), (1b), (1c), except that the model intercept $\beta_{\mathrm{US}}$ represents the average slope in US areas, and the contrast $\delta_{\beta }$ represents the difference in average slope between Albertan and US areas, with $\phi_{\mathrm{AB}}$ representing an indicator variable ($\phi_{\mathrm{AB}}=0$ for US areas, $\phi_{\mathrm{AB}}=1$ for Albertan areas); $\sigma_{\mathrm{loc}}^{2}$ is residual variances, representing variability among areas within the US and Alberta. Across all measures of horn size and size at age, estimates from the model in Equation (3a), (3b), (3c) suggest that trends are more negative in Alberta than throughout the United States. Particularly for those measures that are most relevant to evolution (size corrected for age), and when considering those areas with stable size‐based harvest regulations, most of the posterior distribution of the $\delta_{\beta }$ is negative, indicating substantial statistical support for LaSharr et al.'s hypothesis that patterns consistent with evolution of slower horn growth should be stronger in Alberta than elsewhere.

## 4 BIAS ASSOCIATED WITH A FIXED THRESHOLD FOR HARVEST

The magnitude of trends in horn size is downwardly biased in data from animals harvested under a strict phenotype‐based threshold. This phenomenon has been demonstrated thoroughly by Pelletier et al. (2012) and Festa‐Bianchet et al. (2015). More generally, it is well established that size‐dependent mortality and harvest require careful consideration when estimating growth (Ricker, 1969). In this section, we attempt to make the most important considerations as clear as possible.

**FIGURE 2:**
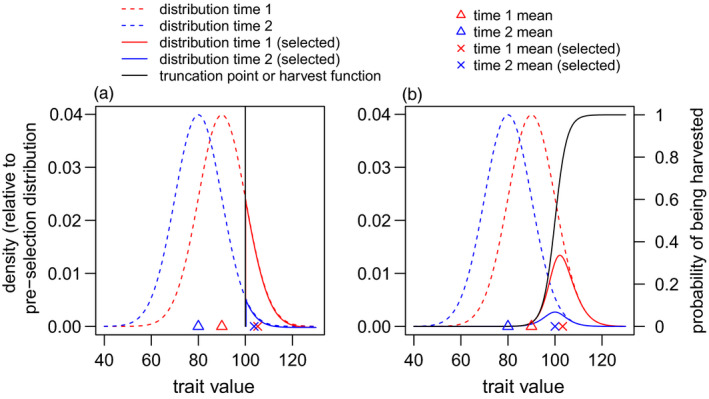
Bias in estimating trends in mean phenotype from highly selected data. (a) depicts the change in the mean of a truncated normal distribution as a function of the underlying (nontruncated) normal distribution. (b) depicts a situation when less severe selection than truncation is applied, in the form of a logistic survival function

Consider the phenotypic distributions depicted by red and blue dotted lines in Figure 2a. These distributions differ in mean by 10 cm. Imagine that the phenotypic distributions represent a population where the mean phenotype declined from 90 cm (red) to 80 cm (blue). Now, imagine that the only data available are from individuals with phenotypes greater than 100 cm (the threshold in Figure 2a). Would the available data from the two time periods estimate the magnitude of the change?

The mean of a truncated normal distribution, $\mu_{t}$, is given by

(4) $$\mu_{t}=\mu +\sigma^{2}\frac{\phi \;(t,\mu ,\sigma^{2})}{1‐\Phi \;(t,\mu ,\sigma^{2})},$$

where μ and $\sigma^{2}$ are the mean and variance of the (nontruncated) normal distribution, and ϕ() and Φ() are Gaussian probability density and cumulative density functions, respectively. Our qualitative conclusion is not dependent on the trait having a normal distribution, but analytical results for the normal distribution illustrate it. The mean of the truncated red distribution is 105.25 cm, and the mean of the blue is 103.73 cm. A true decline of 10 cm is underestimated by −6.6. This phenomenon is not restricted to threshold‐based selection. Figure 2b shows a highly selective logistic function. If this function describes the probability of being harvested, then the corresponding distributions of available data are given by the solid red and blue lines. In the numerical example in Figure 2b, the true change in phenotype of 10 cm is reflected by a change of about −3.3 cm in the measured sample.

**FIGURE 3:**
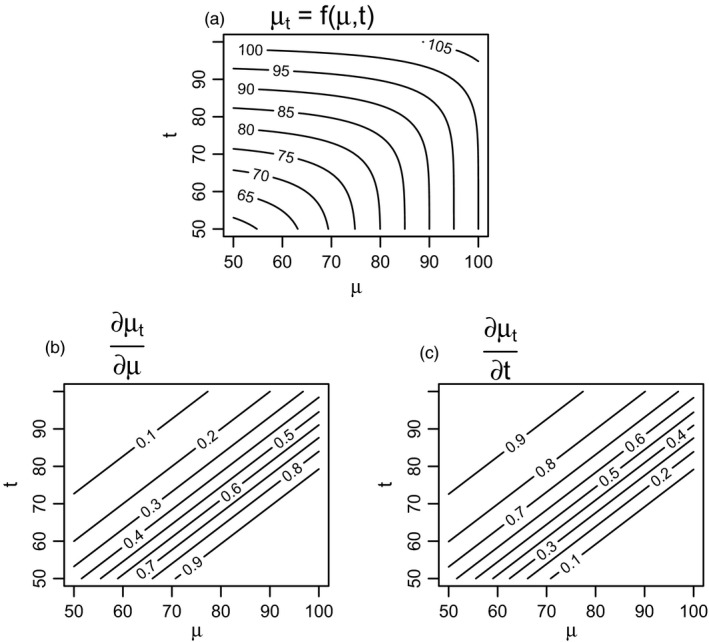
The mean of a truncated distribution can be determined much more by the truncation point than by the mean of the underlying distribution. (a) mean of values of a truncated normal distribution, $\mu_{t}$ with underlying means μ betwen 50 and 100, truncated to include only values exceeding t, with t between 50 and 100; the function plotted in (a) is that in Equation (4). When the truncation point is greater than the mean, the mean of the underlying distribution has far less influence on the mean of the truncated distribution than does the truncation point. In (a), contours of $\mu_{t}$ tend towards horizontal lines when $t>\mu_{t}$, in the upper left corner. The key conditions where t primarily determines values of $\mu_{t}$ are shown more directly by the partial derivatives of $\mu_{t}$ with respect to μ and t, in plots (b) and (c), respectively. When $t>\mu_{t}$, $\frac{\partial \mu_{t}}{\partial \mu }$ takes large values, approaching one (c), while $\frac{\partial \mu_{t}}{\partial \mu }$ takes small values (b), approaching zero

While $\mu_{t}$ is entirely determined by the difference between the truncation point and the underlying mean, that is, by t‐μ, for any given variance, it is useful to visually examine Equation (4), and the partial derivatives of $\mu_{t}$ with respect to both μ and t. Figure 3 shows how, when t‐μ is large, $\mu_{t}$ is almost entirely influenced by the truncation point, t, almost without any effect of the true underlying mean. A more explicit treatment of how the mean of a truncated distribution can depend more on the truncation point than on the true mean is given by the derivatives of $\mu_{t}$ with respect to the underlying mean μ and the truncation point t. For changes in the mean of a truncated distribution to be indicative of changes in the underlying mean, $\frac{\partial \mu_{t}}{\partial \mu }$ would ideally be near one. For a large portion of the parameter space (when t‐μ is large, corroborating the interpretation of Figure 3a), $\frac{\partial \mu_{t}}{\partial \mu }$ is much less than one, while $\frac{\partial \mu_{t}}{\partial \mu }$ is larger, and closer to one, indicating that any trends (or stability) is likely to be more reflective of changes (or stability) in the truncation point.

Equation (4) contains useful biological lessons. First, one should be extremely careful when using truncated data. Means in truncated data can have virtually no bearing on the underlying mean of the distribution. Equation (4) also tells us when the mean of a truncated distribution will be most associated from the true mean. When much of the distribution is above the truncation point, its mean reflects the true mean. However, if much of the underlying distribution is below the truncation point, then almost nothing can be said about the true mean. As such, when size‐based regulations are the primary determinant of harvests, or minimum trophy scores are the key criterion for inclusion in record books, the resulting data may have very little bearing on population means.

LaSharr et al. (2019) present simulations that purport to contradict the general conclusion that threshold‐based data collection reduces the ability to characterize trends in a phenotype. In contrast to a threshold for harvest based on the degree of horn curl, which will be closely determined by horn length, LaSharr et al. (2019) define a “moving” threshold with reference to the existing mean at any given time. To simplify the situation to truncation of a normal distribution, to generate a tractable and informative analytical model (a basic feature of the LaSharr et al., 2019 model), we can modify Equation (4) by noting that a truncation point that is defined relative to the mean τ can be related to a fixed truncation point t, as defined above, as t=τ+μ. The mean of a truncated distribution in terms of this truncation point τ that is defined relative to the mean is then

(5a) $$\mu_{\tau }=\mu +\sigma^{2}\frac{\phi \;(\tau +\mu ,\mu ,\sigma^{2})}{1‐\Phi \;(\tau +\mu ,\mu ,\sigma^{2})},$$

(5b) $$=\mu +\sigma^{2}\frac{\phi \;(\tau ,\;0,\;\sigma^{2})}{1‐\Phi \;(\tau ,\;0,\;\sigma^{2})}$$

So, the mean of the truncated distribution is given by the underlying mean, plus a quantity $\sigma^{2}\frac{\phi \;(\tau ,0,\sigma^{2})}{1‐\Phi \;(\tau ,0,\sigma^{2})}$. This additional quantity does not depend on the mean, and so $\frac{\partial \mu_{\tau }}{\partial \mu }=1$. As such, a change in $\mu_{\tau }$ is perfectly reflective of a change in μ, when a “moving” truncation point is defined relative to the mean itself.

A moving truncation point, defined along the lines of τ, could be a rough model of selection induced by hunters, when they are presented with abundant legally harvestable animals. Such a pattern of selectivity will certainly contribute to the more complex overall picture of data resulting from measurements of trophies. When a moving threshold is a good model of nonrandom harvests, the harvest data could be used to make robust inference about trends.

The most pervasive feature of the hunt areas (all those in Alberta) that LaSharr et al. (2019) characterize as having management practices likely to create the strongest selection, is a scarcity of legal rams, combined with a fixed threshold. In Alberta, where most hunt areas apply fixed thresholds and unlimited licences, any moving threshold is likely to be a very poor reflection of the nature of phenotype‐based harvest and thus data collection. In contrast, few jurisdictions in the United States use threshold‐based harvest regulations, and those that do also limit harvest so that LaSharr et al.'s (2019) may be reasonably representative.

This section considered the mean of a distribution truncated at a single time point. In a long‐lived animal, the consequences of truncation will be more complicated. As a cohort matures, and its horns grow, selection of horn size by hunters, and thus the accumulation of data from trophies, will occur over several years. Phenotypic changes will affect the age‐specific probability of exceeding the truncation threshold. The mathematical study of truncation in this section is aimed at understanding, in isolation, how truncation contributes to a more complex data generating mechanism.

## 5 ADDITIONAL BIAS ASSOCIATED WITH CHANGING AGE STRUCTURE

The interaction of threshold‐based harvest and age structure increases the scope for biases that would dampen detection of temporal trends in horn size. Two consequences of age structure are relevant. Within a cohort, faster growing individuals will be harvested at younger ages than slower growing individuals (Douhard et al., 2016; Hengeveld & Festa‐Bianchet, 2011). The fastest growing individuals will be harvested at relatively young ages, and the mean size at age of individuals harvested at older ages will be downwardly biased. As a result, growth rates estimated from harvest data will be downwardly biased. The underlying principles are well established (Ricker, 1969), and we illustrate them here with the help of a numerical toy example aimed qualitatively at the application to horn growth.

When the downward bias in estimated growth rate is combined with the expectation that mean age at harvest will increase if growth rates decline, as reported by Festa‐Bianchet et al. (2014), then an additional bias will arise. LaSharr et al. (2019) predict mean size at age seven from growth functions estimated from harvest data. Since these growth functions are too shallow, they underestimate the mean size at age seven of individuals that are harvested relatively young, and overestimate the mean size at age seven of individuals shot at relatively old ages. Where horn growth rates have declined, such that harvested individuals show an increasing age trend, the change detected from trophy harvests will be further dampened because older individuals will have upwardly biased estimates of mean size at age seven.

There are too many unknowns to quantify the degree to which bias generated by the combination of underestimated growth rates and shifting mean age at harvest influence results in LaSharr et al. (2019). We will therefore only illustrate the qualitative bias. We simulated three cohorts with growth mean rates of $\bar{g}=\left[0.8,1.0,1.2\right]$. We simulated 1000 individuals in each cohort and assigned each individual i a growth rate effect described by a standard random deviate $\zeta_{i}$. We then assigned each individual a horn size trajectory, relative to a harvest threshold with an arbitrary value of zero, using the formula $y_{\mathrm{ia}}=‐6+\left(\bar{g}+\zeta_{i}\right)a$ for integer values of age, a, between four and 10. In the growth expression, −6 is an initial size, relative to the threshold for harvest (and therefore measurement) that we arbitrarily set to zero; these only have relevance with respect to each other, and their exact values are determined by convenience for plotting. We simulated harvest by selecting individual records of size at age, if size at age exceeded the threshold, with probability 0.5, simulating a 50% annual harvest rate of harvestable males.

**FIGURE 4:**
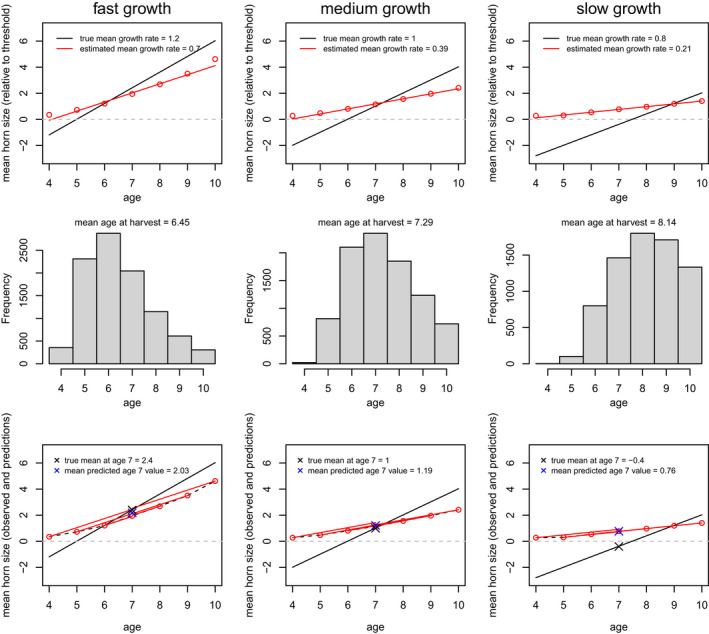
Bias arising from the interaction of phenotype‐dependent harvest and shifting age structure of harvested individuals. Columns from left to right simulate cohorts with decreasing growth rates. The top row shows true growth rates (solid lines) and growth rates estimated from harvest data (red lines). The middle row shows the shifting age structure of harvested individuals, as growth rate declines but the threshold for harvest remains the same. The bottom row shows the prediction of mean size from the biased estimated growth functions. Predicted values at the standard age (seven) are most upwardly biased when the growth rate is lowest, generating a bias that will dampen any trend for decreasing horn size. In the bottom row, red points, connected with lines, show mean size at harvest for each age, connected to the mean predicted size at age 7

We recorded the trajectory of size at age in each cohort, and the trajectory inferred from size at age of harvest (top row of Figure 4). Mean growth rates were underestimated by harvested individuals across all cohorts. The slowest‐growing cohort had a 73% downward bias, relative to approximately 42% bias in the fastest growing cohort. Age at harvest increased from the fastest to the slowest‐growing cohort (middle row of Figure 4). Truncation combined with the changing age structure yielded the most severe upward bias of predicted sizes at age seven in the cohort with the lowest growth rate (bottom right panel of Figure 4), demonstrating a further bias in inference of trends in growth rate or size at age from harvest data.

## 6 DISCUSSION

Data from harvested animals can give biased inferences of temporal changes in growth rates when harvest is based on a fixed threshold. Despite multiple biases that will dampen any trends for decreases in growth rate, if and where they exist, the LaSharr et al. (2019) data indicate a much more widespread declining trend in bighorn sheep horn growth rates than their conclusions reflect. LaSharr et al.'s (2019) conclusion that horn sizes in Alberta are stable in most locations relies on interpreting nonsignificant trends as evidence for lack of a trend. Using meta‐analysis, we estimated the average trend and the heterogeneity of trends across management areas in Alberta (Tables 1 and 2, Figure 1). The LaSharr et al. (2019) results thus indicate a consistent pattern of decline across Alberta, with regulations based on a fixed threshold to define legally harvestable rams, combined with no limit on harvest, is expected to generate the strongest selection on horn size.

**TABLE 2:** Differences in temporal trends of mean horn size of harvested bighorn sheep between Albertan and US areas based on the model given in Equation (3a), (3b), (3c), wherein the term $\delta_{\hat{\beta }}$ represents the difference between trends in Alberta vs the United States

Management area	$\delta_{\bar{\beta }}$	$P\left(\beta_{\mathrm{US}}‐\beta_{\mathrm{AB}}<0\right)$	$P(H_{0}:\beta_{\mathrm{US}}=\beta_{\mathrm{AB}})$
(a) All areas			
Size	−0.03 (−0.13 to 0.08)	0.709	0.582
Size, corrected to age 7	−0.06 (−0.11 to −0.01)	0.988	0.024
Size, corrected to age 7 and controlling for environment	−0.05 (−0.11 to 0.00)	0.973	0.054
(b) All areas with consistent regulations^a^			
Size	−0.06 (−0.14 to 0.02)	0.936	0.128
Size, corrected to age 7	−0.07 (−0.12 to −0.01)	0.994	0.012
Size, corrected to age 7 and controlling for environment	−0.06 (−0.11 to −0.01)	0.986	0.028

Note

Estimates of $\delta_{\hat{\beta }}$ are reported with 95% credible interals, and also $P(\beta_{\mathrm{US}}‐\beta_{\mathrm{AB}}<0)$, the proportion of posterior distribution of the $\delta_{\hat{\beta }}$ that is negative (indicating more negative trends in Alberta than elsewhere), and $P(H_{0}:\beta_{\mathrm{US}}=\beta_{\mathrm{AB}})$ the two‐sided quasi p value associated with the null hypothesis of equal mean slopes in Alberta and in the United States.

^a^ One management area in Alberta is expected to have a substantially positively biased trend as a result of a change to the size‐based harvest regulations during the course of the study and is excluded from analyses reported in part (b). This bias is likely to be manifested primarily in the raw size data.

Because of the biases inherent to phenotype‐based data collection, it would be unwise to attempt to interpret the magnitude of the declines in horn size throughout Alberta. However, it seems inescapable that those magnitudes are underestimated. We have elucidated two mechanisms – truncation (Figures 2 and 3), and changes in age structure (Figure 4) – that likely bias estimates of the magnitude of changes in mean horn size, as inferred from trophy data, towards smaller values.

Documentation of temporal trends in mean phenotype is a weak form of inference of evolutionary change (Endler, 1986). LaSharr et al.'s (2019) analysis also attempts to control for changes in environmental variables. Insofar as such an analysis can account for changes in the environment to which phenotypes may respond plastically, any remaining temporal trend is much more reasonably interpreted as an evolutionary change. However, any environmental changes beyond those captured by the available environmental data will contribute to temporal trends in phenotype, concomitant with any evolutionary change. We therefore caution against interpretations of changes in mean phenotype, even accounting for environmental covariates at the enormous spatial scale of LaSharr et al.'s (2019) analysis, as strong evidence for evolutionary change. Associations between trends in phenotypes and selective pressures are an additional type of evidence for an evolutionary response to natural selection (Endler, 1986). The evident pattern in LaSharr et al.'s (2019) results (Tables 1 and 2, Figure 1) of much stronger trends in phenotype in Alberta than elsewhere correlates with the expected strength of selection. Thus, insofar as the data reported in LaSharr et al. (2019) can provide evidence for or against responses to anthropogenic selection, and they clearly support the possibility that evolution of smaller horns has occurred where selection is expected to be strongest. Given the biases in the underlying data, the magnitudes of the trends resulting from LaSharr et al.'s (2019) analysis should be seen as a lower limit on declines that have occurred.

## CONFLICT OF INTEREST

The authors declare no conflict of interest.

## Data Availability

All data are publicly available in the Supporting information of LaSharr et al. (2019).
